# A biological function based biomarker panel optimization process

**DOI:** 10.1038/s41598-019-43779-2

**Published:** 2019-05-14

**Authors:** Min Young Lee, Taek-Kyun Kim, Kathie-Anne Walters, Kai Wang

**Affiliations:** Institute for Systems Biology, Seattle, Washington United States of America

**Keywords:** Biomarkers, Biomarkers

## Abstract

Implementation of multi-gene biomarker panels identified from high throughput data, including microarray or next generation sequencing, need to be adapted to a platform suitable in a clinical setting such as quantitative polymerase chain reaction. However, technical challenges when transitioning from one measurement platform to another, such as inconsistent measurement results can affect panel development. We describe a process to overcome the challenges by replacing poor performing genes during platform transition and reducing the number of features without impacting classification performance. This approach assumes that a diagnostic panel reflects the effect of dysregulated biological processes associated with a disease, and genes involved in the same biological processes and coordinately affected by a disease share a similar discriminatory power. The utility of this optimization process was assessed using a published sepsis diagnostic panel. Substitution of more than half of the genes and/or reducing genes based on biological processes did not negatively affect the performance of the sepsis diagnostic panel. Our results suggest a systematic gene substitution and reduction process based on biological function can be used to alleviate the challenges associated with clinical development of biomarker panels.

## Introduction

Multi-gene biomarker panels identified from gene expression data can be used to diagnose diseases and/or stratify patients into different disease stages. Machine learning and data mining algorithms have been adapted to identify biomarker candidates. The general workflow for transcript-based biomarker panel discovery includes identifying disease-affected genes (e.g. differentially expressed genes), selecting a subset of genes (features) for the panel, and building a classifier (decision rule) based on the selected features. Various feature selection methods (recursive feature elimination (RFE), least absolute shrinkage and selection operator (LASSO) method, and the Boruta and Vita approaches) have been used^1–3^ by considering feature decencies and interaction with classification methods such as support vector machine (SVM), logistic regression (LR), and random forest (RF) that have been widely applied to build diagnostic panels to discriminate between states^4^. Based on this general approach, many biomarker panels have been reported in the literature but few have been successfully developed into clinical assays^5,6^. In part, this is due to the technical difficulties during the transition from discovery to development phase. To implement transcript based biomarkers that were identified from high-throughput transcriptomic data such as microarray or next generation sequencing (NGS), the transcripts (features) need to be measured with a platform that is more suitable for use in a clinical setting such as quantitative polymerase chain reaction (qPCR)^7,8^. However, there are technical challenges when moving from high-throughput platforms into different measurement methods like qPCR. A major issue is the inconsistency of results between different measurement platforms^9–11^. It is also a challenge to develop a panel with 30 or 40 features for clinical use due to cost and implementation^12^.

To identify a candidate biomarker panel with a limited number of features, Top Scoring Pair (TSP) is potentially useful^13^. The goal of TSP is to identify a pair of genes where the ordering (i.e. the relative expression) of two genes is reversed between groups. TSP is then extended to *k*TSP, *k* pairs of genes, to achieve a higher performance through additional pairs of genes^14^. To address the inconsistency between measurement platforms^15^, demonstrated that poor performing features (genes) in a 17 gene panel for predicting clinical outcome of breast cancer patients can be substituted with highly correlated genes in the same pathway^15^. Additionally, the authors showed that the number of features in the panel can be reduced, although the original 17 gene panel remained the best predictor.

In this study, we describe a systematic process to optimize transcript based biomarker panels by both replacing poor performing genes (features) and reducing the number of features in the panel. The approach is based on the similar hypothesis described by Liu *et al*. in that a diagnostic panel measures the combined effect of dysregulated biological processes during the development of a disease and that highly correlated genes involved in the same biological processes should share similar discriminatory power. We further filtered substitution candidates by selecting those that have the same directional changes of its concentrations in the disease, rather than simply the highest correlated gene. The complex regulatory network in a biological system coordinately affects multiple genes relating to the phenotype that can be equivalent to each other for discriminating the phenotype^16^. This biological function-based gene substitution provides a solution for gene measurement issues encountered during platform transition. Our approach can also be used to reduce the features in a diagnostic panel by identifying a minimal set of genes to represent core biological processes in a multi-gene biomarker panel.

The utility of the biomarker optimization process was demonstrated using a published sepsis diagnostic panel, referred to here as the Stanford11 gene panel^17^. We investigated (1) critical biological pathways associated with the Stanford11 gene panel, (2) the impact of gene substitution on the diagnostic performance of the Stanford11 gene panel, and (3) minimal core genes required for the diagnostic panel. We showed that substitution and reduction of more than half of the Stanford11 does not affect the performance. Multiple alternative panels with less features compared to the Stanford11 were identified, but have equivalent diagnostic performance. The results suggest a systematic gene substitution and reduction process can be used to alleviate the challenges associated with translation of candidate biomarker panels into clinically applicable assays without sacrificing the performance of a biomarker panel.

## Results

### Identification of substitutable genes for the Stanford11 sepsis diagnosis panel

**Figure 1:**
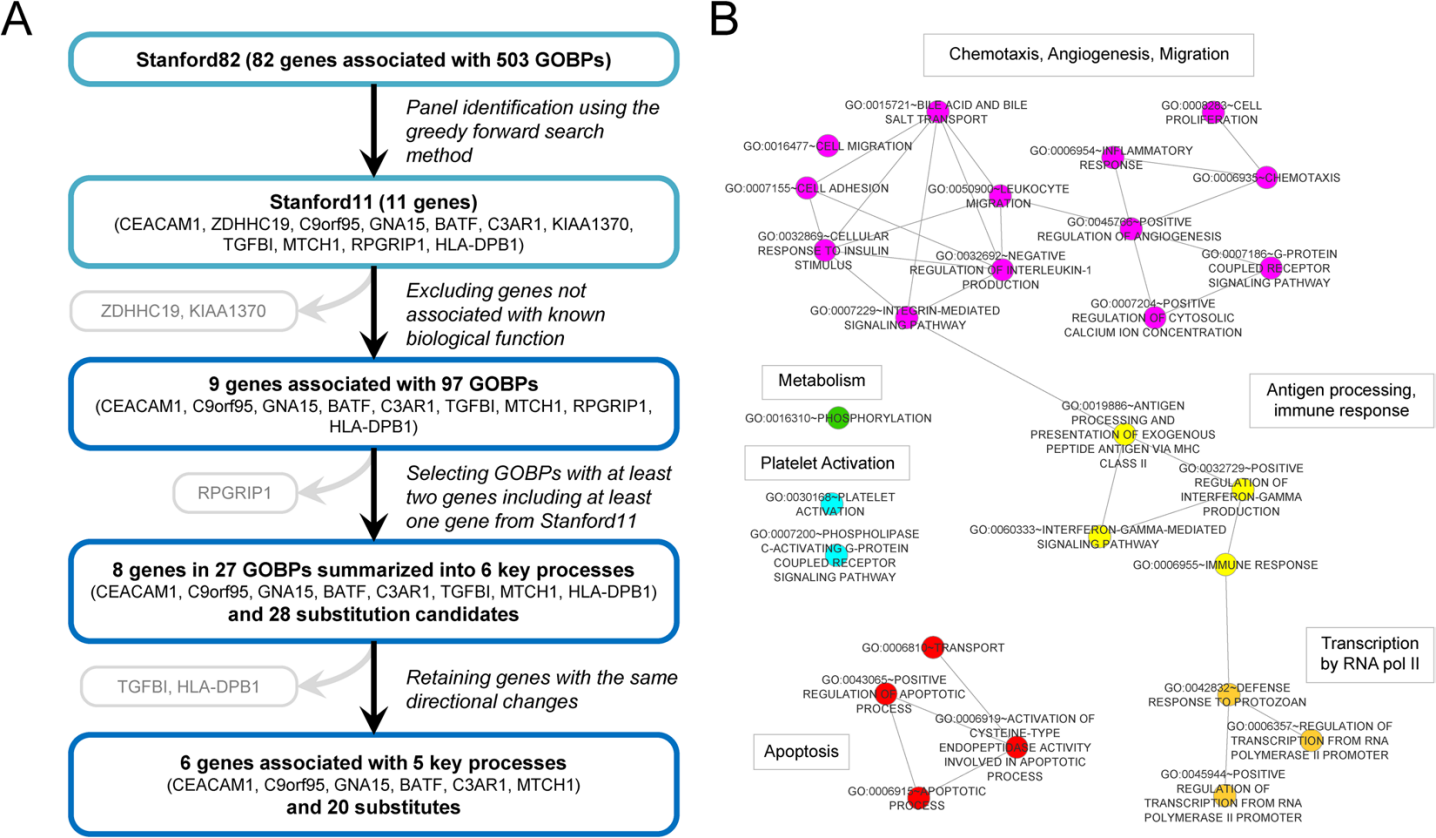
Identification of substitutes for Stanford11. (**A**) The overall procedure of the identification of substitutes for Stanford11. (**B**) The six key biological processes represented by the Stanford11 panel.

The overall procedure and the results of identifying substitutable genes for Stanford11 are summarized in Fig. 1A. There were 503 gene ontology biological processes (GOBPs) represented by at least one of the 82 differentially expressed genes (DEGs) termed Stanford82. The genes in the Stanford11 panel were involved in 97 different GOBPs, with the exception of ZDHHC19 and KIAA1370 which are not associated with known biological function. Among the 97 GOBPs associated with Stanford11, 27 of them had at least one additional gene from the Stanford82 gene pool that is not in the Stanford11 panel and can be used as potential substitution candidates. These 27 GOBPs are represented by six key biological processes; (1) chemotaxis, adhesion, migration; (2) antigen processing, immune response; (3) transcription by pol II; (4) platelet activation; (5) apoptosis; and (6) metabolism (Fig. 1B). We summarized substitution candidates that are involved in these 6 key biological processes associated with the Stanford11 panel (Table 1). Among the six key biological processes, two processes, chemotaxis, adhesion, migration and platelet activation, have more than two genes in the panel and the remaining four processes have just one gene. RPGRIP1, a gene associated with neural precursor cell proliferation and retina development, is substitutable only with the genes already included in the Stanford11 panel and so was excluded for gene substitution. Among the substitutable genes, only genes with the same directional changes as genes in the Stanford11 panel, i.e., increased or decreased expression level in sepsis patients compared to controls were selected. Among the 28 substitution candidates, 20 genes were retained for six genes in the Stanford11 panel (CEACAM1, C3AR1, GNA15, BATF, MTCH1, and C9orf95) representing five biological functions. TGFBI (GOBP chemotaxis, angiogenesis, adhesion, migration) was removed since no substitutable gene with the same directional change was retained. There was also no substitutable gene for HLA-DPB1 (immune response function); therefore, HLA-DPB1 was retained to keep all six biological processes during substitution and reduction procedures. Interestingly, most of the six genes that can be substituted in the Stanford11 panel showed increased expression level in sepsis patients except MTCH1, a gene involved in apoptosis (Table 1).

**Table 1:** The list of substitutable genes for features in the Stanford11.

	Chemotaxis, adhesion, migration	Antigen processing, immune response	Transcription by RNA pol II	Platelet activation	Apoptosis	Metabolism
CEACAM1↑	ADAMTS3↑, CCR1↑, CD177↑, CD63↑, EMR1↑, FCER1G↑, IL10↑, OSTalpha↑, PSTPIP2↑, SIGLEC9↑, TBC1D4↓					
C3AR1↑	ANXA3↑, CCR1↑, CD177↑, EMR1↑, FCER1G↑, FES↑, FFAR3↑, IL10↑, S100A12↑			GPR84↑		
TGFBI↓	CCR1↑, EMR1↑, FES↑, SIGLEC9↑					
GNA15↑	CCR1↑, EMR1↑, FFAR3↑			FCER1G↑, P2RX1↑		
HLA-DPB1↓		BPI↑, CCR1↑, FCER1G↑, FCGR1B↑, IL10↑, IL18R1↑				
BATF↑			IL10↑, PLAC8↑, GLO1↓, PRKRIR↓, WDR75↓			
MTCH1↓					LCN2↑, OSTalpha↑, P2RX1↑, ARHGEF18↓	
C9orf95↑						C9orf103↑, SEPHS2↑

Substitution candidates (n = 28) for eight genes of Stanford11 were selected based on six key biological processes. ↑ And ↓ indicates increased or decreased expression level in sepsis, respectively. No substitutable gene was retained for TGFBI and HLA-DPB1 after considering consistency in directional changes.

Since three genes (ZDHHC19, KIAA1370, and RPGRIP1) were excluded during the process of identification of substitutable genes, their contribution to the classification performance (defined as area under the curve, AUC) of Stanford11 was assessed. Interestingly, excluding these genes from Stanford11 did not significantly affect the performance in both discovery and validation sets (Supplementary Table S3). In the case of excluding all three genes, the performance decreased in one discovery and one validation set (Glue grant day [1–3] from 0.9145 to 0.865 and GSE74224 from 0.8814 to 0.8544) while increasing in one discovery set (GSE40012 from 0.7091 to 0.774). Therefore, the three genes contributed marginally to the diagnostic performance of Stanford11.

### Importance of biological processes on classification performance

**Figure 2:**
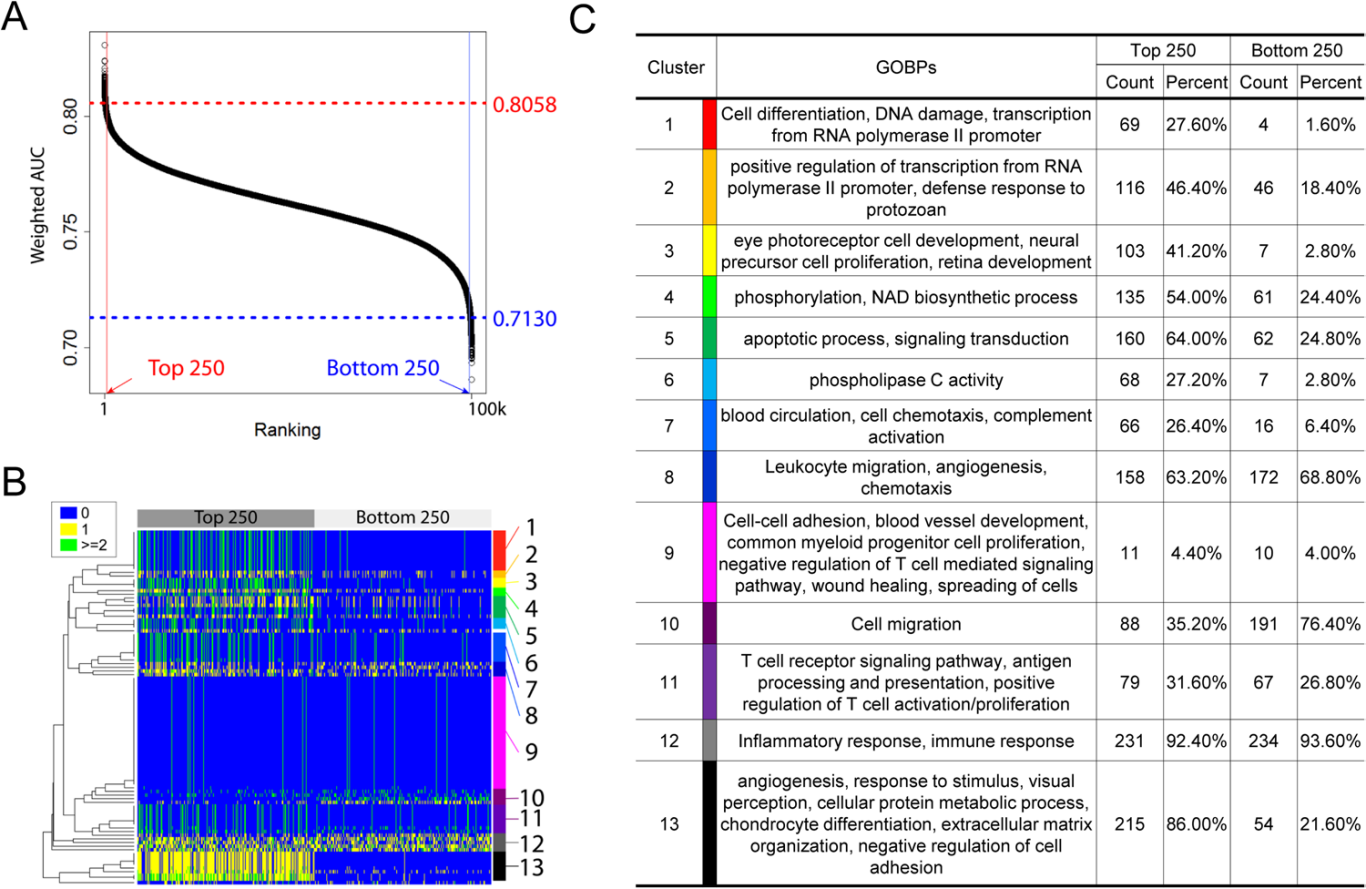
Importance of biological processes on classification performance. (**A**) The distribution of classification performances of the 100,000 random gene sets. (**B**) The number of genes of the top and bottom 250 gene sets in 97 GOBPs represented by Stanford11. (**C**) Clusters of GOBPs in the top 250 and bottom 250 gene sets. Count and percent indicate the average number and percentage of genes in each GOBP cluster.

To determine the impact of biological processes in the performance of a diagnostic panel, we generated 100,000 panels consisting of 11 genes randomly selected from the Stanford82 list. The classification performances of the 100,000 random 11 member gene sets in the nine discovery datasets were computed and sorted based on the performance as measured by AUC (Fig. 2A). The GOBP associated with genes in the top 250 and bottom 250 gene sets were summarized in Fig. 2B. The GOBPs represented by the top 250 gene sets were similar to the Stanford11 six key processes (Fig. 2B), such as transcription by pol II (cluster 1 and 2 in Fig. 2B,C), phosphorylation (cluster 4), apoptosis (cluster 5), PLC (cluster 6), chemotaxis (cluster 7), antigen processing and presentation (cluster 11), metabolic process (cluster 13). Interestingly, the neural precursor cell proliferation and retina development process in cluster 3 was also frequently involved in high performing gene sets. RPGRIP1, which has no substitutable gene, was involved in this biological process. However, removing RPGRIP1 from Stanford11 did not significantly decrease diagnostic performances as shown in Supplementary Table S3. It suggests that genes involved in this biological process have discriminating power but are not essential to retain classification performance of gene panels.

### Impact of gene substitution on classification performance

A total of 12 microarray datasets from the public domain were used to evaluate the effect of gene substitution/reduction on the diagnostic performances of Stanford11. Nine of the datasets were used in the original discovery of the Stanford11 gene panel. The other three, GSE65682, GSE74224 and E-MTAB-3589, were not used in the development of Stanford11 panel; therefore, they were used as independent validation data in this study.

**Figure 3:**
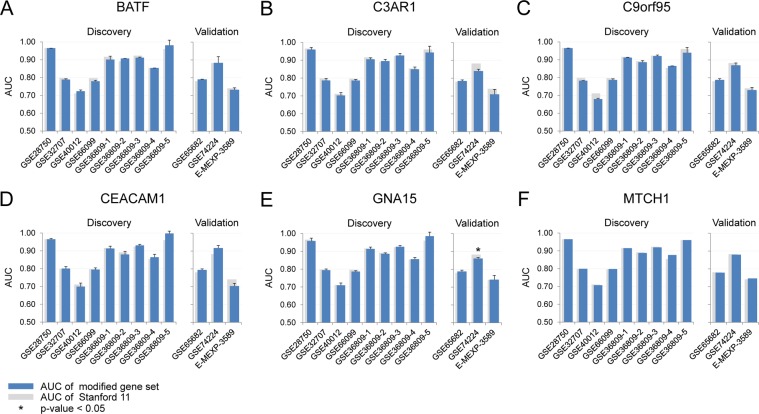
The performances of one gene substitution. The distribution of AUCs in the 12 microarray datasets when (**A**) BATF; (**B**) C3AR1; (**C**) C9orf95; (**D**) CEACAM1; (**E**) GNA15; (**F**) MTCH1 was replaced with a substitute gene. *Indicates P-value from DeLong test comparing a substituted panel (blue bars) with the median AUC and the original Stanford11 (gray bars) less than 0.05.

Based on the substitution candidates listed in Table 1, we changed one gene at a time for the six substitutable genes in the Stanford11 panel. As shown in Fig. 3A–F, one gene substitution does not affect the overall diagnostic performance in both the nine discovery and three validation datasets. In discovery datasets, the average AUCs of the substitutions were not significantly lower than the original Stanford11 panel except using the GSE74224 dataset when replacing GNA15 (Fig. 3E).

**Figure 4:**
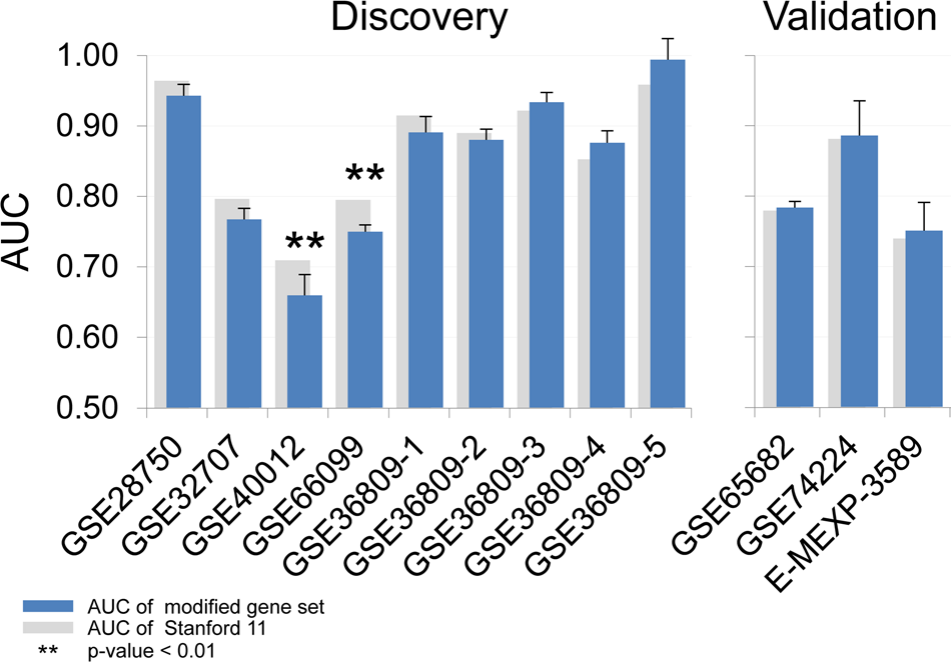
The performances of five gene substitution. The AUCs in the 12 microarray datasets when genes representing all five functional categories were replaced with substitute genes. *and **indicate P-value from DeLong test comparing a substituted panel with the median AUC and the original Stanford11 less than 0.05 and 0.01, respectively.

We then tested the effect of substituting genes representing all functional categories simultaneously. If a function had more than one substitutable gene, all combinations of genes were enumerated and their classification performances were tested and summarized in Fig. 4. Except for the GSE40012 and GSE66099 datasets used in the Stanford11 discovery process, there were multiple five gene substitutions that showed similar performances as the original Stanford11. In summary, gene substitution based on the same biological process and direction of concentration changes can provide alternative panels that have similar diagnostic performance.

### Impact of gene reduction on classification performance

**Figure 5:**
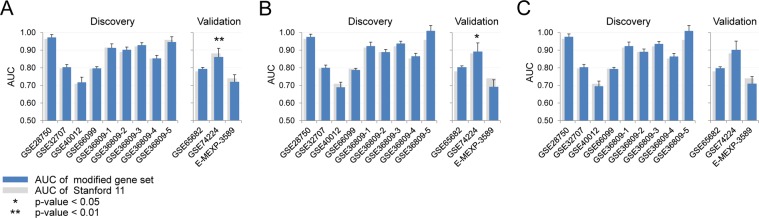
The performances of gene reduction. The AUCs in the 12 microarray datasets when only one gene in (**A**) platelet activation function; in (**B**) chemotaxis, adhesion, migration function; (**C**) in both processes was retained. *and **indicate P-value from DeLong test comparing a substituted panel with the median AUC and the original Stanford11 less than 0.05 and 0.01, respectively.

The possibility of using representative genes from each biological process to reduce the number of features in the diagnostic panel was investigated. Among the six representative biological processes of the Stanford11 panel, two biological functions have more than one gene in the panel. The GOBP, “platelet activation function” has two genes (C3AR1 and GNA15) and “chemotaxis, adhesion, migration function” has four genes (CEACAM1, C3AR1, TGFBI, and GNA15) in the panel. The AUCs of panels where only one gene was retained were calculated. As shown in Fig. 5A, the two panels with only one gene retained from “platelet activation function” have similar performance to the Stanford11. The panels with only one gene from the GOBP “chemotaxis, adhesion, migration function” also have similar performance in the datasets used to identify Stanford11 (Fig. 5B). The impact of retaining only one gene from both GOPBs was tested and the average diagnostic performance was not significantly different from the original Stanford11 panel in all the discovery and validation sets (Fig. 5C). In all cases, there were multiple panels with reduced features that delivered better performances in more than half of the independent validation datasets.

**Figure 6:**
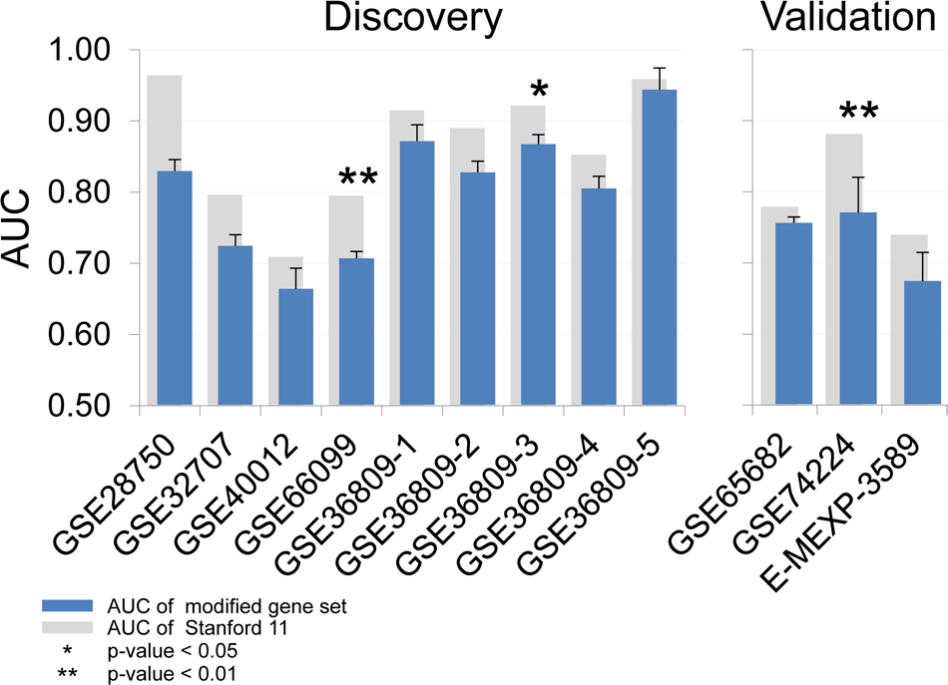
The performances of 1,482 six-gene combinations. *and **indicate P-value from DeLong test comparing a substituted panel with the median AUC and the original Stanford11 less than 0.05 and 0.01, respectively.

To explore the possibility of just using representative genes from key biological processes associated with Stanford11, all possible combinations of six gene panels were generated from genes mapped to those six biological processes. Diagnostic performances for all six-gene combinations were computed (Fig. 6). Among the 1,482 six-gene combinations, 73 new panels which have higher performance than the lower bound of 95% confidence intervals in all discovery datasets than the original panel were selected (Supplementary Table S4). Among the 73 new panels, 22 panels have higher performance even in the two validation sets (GSE65682 and GSE74224, Table 2).

**Table 2:** Optimized six-gene panels (n = 22) with higher performance in the validation sets.

6-gene Panels	Chemotaxis, adhesion, migration	Antigen processing, immune response	Transcription by RNA pol II	Platelet activation	Apoptosis	Metabolism	GSE65682	GSE74224	E-MEXP-3589
1	CCR1	HLA-DPB1	BATF	C3AR1	ARHGEF18	C9orf95	0.7807	0.8854	0.6633
2	CCR1	HLA-DPB1	BATF	C3AR1	MTCH1	C9orf103	0.8170	0.9058	0.6990
3	CCR1	HLA-DPB1	BATF	C3AR1	MTCH1	C9orf95	0.8095	0.8967	0.6786
4	CCR1	HLA-DPB1	BATF	GNA15	MTCH1	C9orf95	0.8080	0.8893	0.6378
5	CD177	HLA-DPB1	BATF	C3AR1	MTCH1	C9orf95	0.8014	0.8827	0.6684
6	CD63	HLA-DPB1	PLAC8	FCER1G	MTCH1	C9orf95	0.8158	0.9128	0.6122
7	CD63	HLA-DPB1	PLAC8	GNA15	ARHGEF18	C9orf95	0.7852	0.9220	0.5663
8	CD63	HLA-DPB1	PLAC8	GNA15	MTCH1	C9orf95	0.8107	0.9333	0.5918
9	CD63	HLA-DPB1	BATF	C3AR1	MTCH1	C9orf95	0.8080	0.9067	0.6633
10	CD63	HLA-DPB1	BATF	GNA15	MTCH1	C9orf95	0.8029	0.8915	0.6378
11	EMR1	HLA-DPB1	BATF	C3AR1	MTCH1	C9orf95	0.8149	0.8963	0.6582
12	EMR1	HLA-DPB1	BATF	GNA15	MTCH1	C9orf95	0.8044	0.8836	0.6071
13	FCER1G	HLA-DPB1	PLAC8	C3AR1	MTCH1	C9orf95	0.8080	0.9241	0.6531
14	FCER1G	HLA-DPB1	PLAC8	GNA15	ARHGEF18	C9orf95	0.7861	0.9098	0.5408
15	FCER1G	HLA-DPB1	PLAC8	GNA15	MTCH1	C9orf95	0.8092	0.9185	0.5816
16	FCER1G	HLA-DPB1	BATF	C3AR1	MTCH1	SEPHS2	0.8086	0.8867	0.6582
17	FCER1G	HLA-DPB1	BATF	C3AR1	MTCH1	C9orf95	0.8071	0.8963	0.6480
18	FES	HLA-DPB1	PLAC8	FCER1G	MTCH1	C9orf95	0.8089	0.8945	0.6173
19	FES	HLA-DPB1	PLAC8	GNA15	ARHGEF18	C9orf103	0.7870	0.9098	0.6122
20	FES	HLA-DPB1	PLAC8	GNA15	MTCH1	C9orf95	0.8005	0.9150	0.6071
21	FES	HLA-DPB1	BATF	C3AR1	MTCH1	C9orf95	0.8026	0.8806	0.6786
22	C3AR1	HLA-DPB1	BATF	GNA15	MTCH1	C9orf95	0.7999	0.9133	0.6480

Among six gene panels (n = 73) that have higher performance than the lower bound of 95% confidence intervals of the original Stanford11 panel in all discovery datasets, 22 panels have even higher performance in two independent datasets.

### Addition of RPGRIP1 to the new six-gene panels

Though removing RPGRIP1 (neural precursor cell proliferation and retina development process) from the original Stanford11 panel did not decrease the overall diagnostic performance (Supplementary Table S3), we tested the effect of adding RPGRIP1 to the new 6 gene panels. The results showed that adding RPGRIP1 to the 6-gene panel only improved the performance in one of the validation datasets, GSE74224, but not in the other two datasets (Supplementary Table S5).

### Evaluation of the impact of biological function information

**Figure 7:**
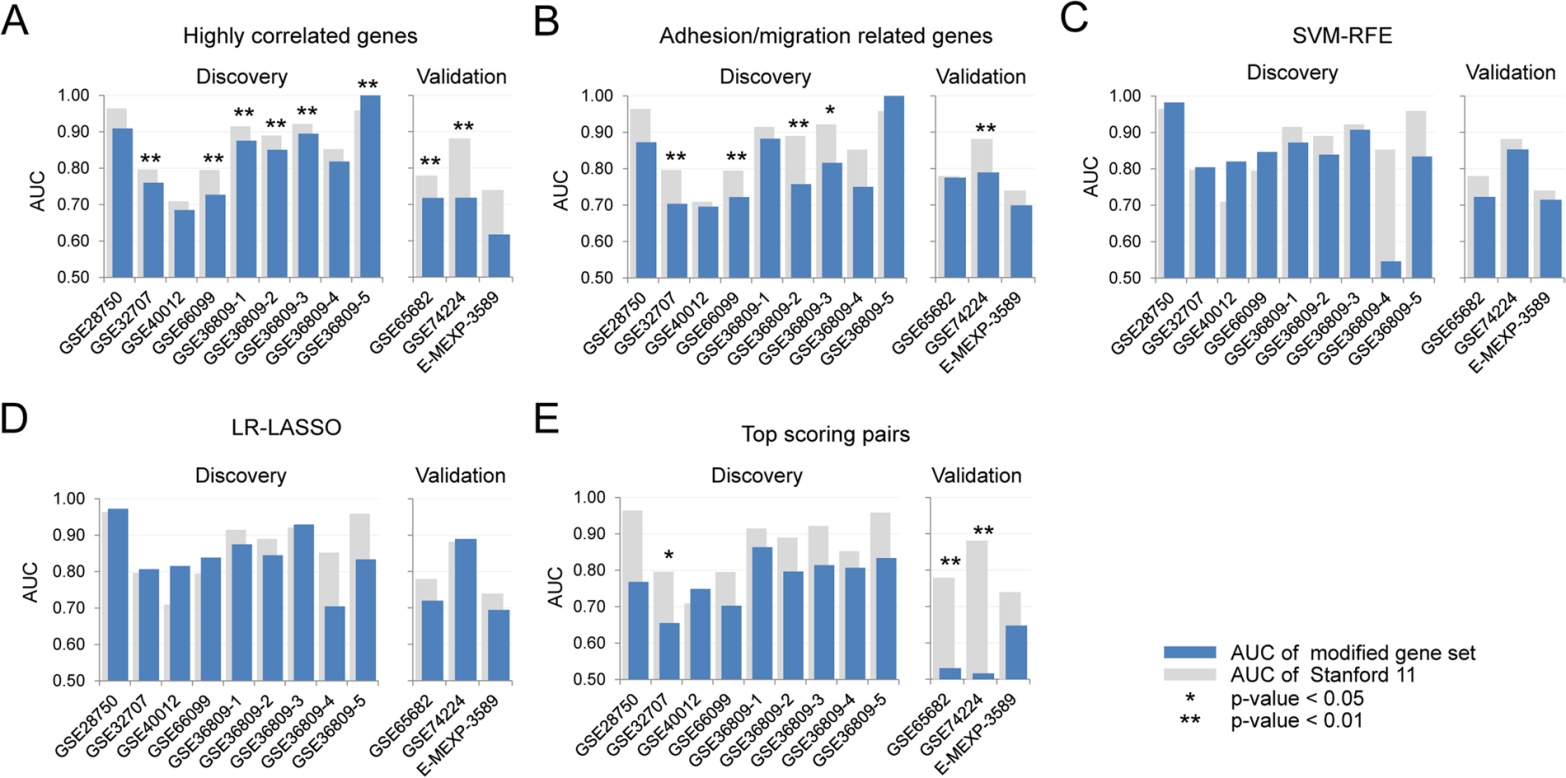
Evaluation of the impact of biological function information. The importance of biological function information was evaluated in five different approaches. (**A**) The AUCs of the Panel-HC. (**B**) The AUCs of the Panel-AM. (**C**) The AUCs of the Panel-SVM. (**D**) The AUCs of the Panel-LR. (**E**) The AUCs of the Panel-kTSP. *and **indicate p-value from DeLong test less than 0.05 and 0.01, respectively.

The performance differences between biological function-based and expression correlation-based substitution were tested. Based on expression profiles of all the discovery datasets, the highest correlated genes with features in the Stanford11 were selected (referred to as Panel-HC) regardless of the biological functions (Table 3). Most of the highest correlated genes with features in the Stanford11 were not from Stanford82 nor involved in the same GOBPs represented by Stanford11. For instance, BATF of Stanford11 is involved in transcription by pol II, but the highest correlated gene, DDAH2 is involved in nitric oxide biosynthetic process. The average AUCs of the Panel-HC in the discovery and validation sets were 0.8238 and 0.6846 which are 3.58% and 14.40% lower than the Stanford11 (average AUC of 0.8544 and 0.7997 in the discovery and validation sets, respectively (Fig. 7A). This suggests maintaining biological processes associated with disease condition is more critical to generating high performance diagnostic panels than maintaining features with highly correlated expression profiles.

**Table 3:** Genes used in evaluation of the impact of biological function information.

The 11 highest correlated genes				Chemotaxis, adhesion, migration processes	SVM-RFE	LR-LASSO	k-Top Scoring Pairs
Stanford11	Panel-HC	CC	Stanford 82	14 genes	10 genes	6 genes	6 genes
BATF	DDAH2	0.7642		C3AR1, CD177, FCER1G, CEACAM1, ADGRE1, CCR1, TGFBI, SIGLEC9, CD63, PSTPIP2, FES, ANXA3, IL10, RETN	BATF, TGFBI, GNA15, C9orf95, MTCH1, C3AR1, ZDHHC19, KIAA1370, RPGRIP1, CEACAM1	BATF, C3AR1, C9orf95, GNA15, MTCH1, TGFBI	TGFBI - C3AR1 GNA15 - CEACAM1 RPGRIP1 - ZDHHC19
C3AR1	SQRDL	0.5393	√				
C9orf95	PTPN22	0.4870					
CEACAM1	GPR84	0.8169	√				
GNA15	FERMT3	0.5874					
ZDHHC19	GPR84	0.7408	√				
HLA-DPB1	HLA-DMB	0.7329					
KIAA1370	KIAA1468	0.6517					
MTCH1	CDK5RAP3	0.6607					
RPGRIP1	NOV	0.5266					
TGFBI	CPVL	0.8078					

√ in Stanford82 column indicates the genes of Stanford82. CC indicates the Pearson’s correlation coefficient between the genes.

By randomly selecting 11 out of 14 genes involved in adhesion/migration process, 364 panels were generated (referred to as Panel-AM) (Table 3). The performances of Panel-AM were also lower than Stanford11 (average AUC of 0.7999 and 0.7546 in the discovery and validation sets, respectively, Fig. 7B) which suggests genes from one biological process do not deliver sufficient diagnostic performance.

We further test the feasibility of using existing algorithms to optimize the panel. Among various wrapper approaches, SVM-RFE and LR-LASSO were applied. Ten (referred to here as the Panel-SVM) and six genes (Panel-LR) were obtained from the Stanford11 genes by the SVM-RFE and LR-LASSO approaches, respectively (Table 3). The classification performance of the two panels was not significantly different from the original Stanford 11 panel (Fig. 7C,D). The SVM-RFE approach did not effectively reduce the number of genes in the panel. The LR-LASSO resulted in a 6 gene panel with similar performance as original Stanford11 panel; therefore, it might be a useful approach to optimize gene panels. However, both approaches can only down-select genes from an existing panel and cannot provide any substitutable genes if any of the original genes fails in assay development process. *k*TSP algorithms were also applied to identify classifiers with a small set of paired genes. It resulted in three pairs which including six genes in total (referred to as Panel-kTSP, Table 3). The Panel-kTSP showed significantly lower performance in independent datasets (AUC < 0.6 and p-value from DeLong’s test of less than 0.01 in two validation data sets in Fig. 7E). Therefore, the *k*TSP approach failed to provide a panel with good classification performance. In addition, similar to the SVM-RFE and LR-LASSO approaches, *k*TSP cannot provide alternative genes.

## Discussion

There are two main considerations when validating and developing a biomarker panel: (1) the measurement inconsistency when transitioning from one measurement platform (for example, microarray) to another (such as qPCR), and (2) the number of features in the panel. Neither revisiting the discovery phase (identifying DEGs, feature selection, and using different computational approaches) to generate new panel(s) nor examining all possible combinations of DEGs already discovered can overcome these challenges. In the case of Stanford11 panel, to identify alternative 11 gene panels from the Stanford82 is computationally challenging, since there are 1.4e + 13 possible combinations of 11 gene panels from the Stanford82 genes. To assess the performance of these panels would take 32,407 days even on a 100 node cluster computer (based on estimated calculation time per one combination for 12 datasets: 0.02 seconds). To address these challenges, we used a systematic process to reduce the number of features and to replace poor performing genes in the panel with other differentially expressed genes with similar biological function. The fact that features are substitutable, based on similar trend of expression changes and biological function, leads to the conclusion that the performance of a biomarker panel has more to do with its connection to disease-perturbed networks than to the specific genes themselves. The result of this feature substitution method is a number of different panels comprising only six genes, with performances at least as high as the original 11 gene panel.

There has been a discussion on the multiplicity of disease signatures^16,18^. Different computational and mathematical algorithms, noisy measurements, and/or different data pre-processing procedures result in different molecular signatures. Some studies also suggested that overfitting and/or artifacts from data analyses also yield different molecular signatures^19^. On the other hand, others have proposed that the multiplicity attributes to the complex regulatory networks of the biological system leading to multiple biomarker sets that are equally predictive. Statnikov *et al*. showed that numerous signatures can be identified regardless of microarray platforms, pre-processing methods, and model algorithms suggesting that intrinsic gene-gene and gene-phenotype relations might be a source of multiplicity^16^. Our results also support this hypothesis by showing that genes involved in the same biological process are interchangeable and share similar discriminatory power.

In conclusion, this study showed a multigene biomarker panel can be optimized through systemic gene substitution and reduction based on biological function information. The method outlined here can be applied to identify alternative panels for other multigene/protein based biomarkers for numerous diseases and may facilitate developing clinically applicable assays by alleviating challenges in transitioning from high-throughput measurement platform to other platforms.

## Methods

### Sepsis MetaScore

The Stanford11 gene panel (CEACAM1, ZDHHC19, C9orf95, GNA15, BATF, C3AR1, KIAA1370, TGFBI, MTCH1, RPGRIP1, and HLA-DPB1) was developed by first identifying 82 differentially expressed genes (DEGs) termed Stanford82 (Supplementary Table S2), from a meta-analysis of nine independent microarray datasets (Supplementary Table S1). The panel was generated using the greedy forward search method, an algorithm to assemble biomarker panels by identifying the best gene to classify samples and sequentially adds the next best genes to increase the classification score until maximum score is achieved. Classification of each sample was determined based on the Sepsis MetaScore (SMS) which was calculated according to the following equation^20^:

$$\begin{array}{c}{\rm{SMS}}=\sqrt[6]{({\rm{CEACAM}}1\times {\rm{ZDHHC}}19\times {\rm{C}}9{\rm{orf}}95\times {\rm{GNA}}15\times {\rm{BATF}}\times {\rm{C}}3{\rm{AR}}1)}\\ \,\,-5/6\sqrt[5]{({\rm{KIAA}}1370\times {\rm{TGFBI}}\times {\rm{MTCH}}1\times {\rm{RPGRIP}}1\times {\rm{HLADPB}}1)}\end{array}$$

SMS is the difference between a geometric mean of up-regulated genes in sepsis and a geometric mean of down-regulated genes in sepsis. The same equation was applied to all alternative panels generated with gene substitution and reduction procedures described below. Classification power of SMS in each dataset was determined by the area under the curve (AUC) of receiver operating characteristic (ROC) curve. Two-sided 95% confidence intervals for AUC were computed using pROC R package^21^. Statistical significance of the difference between AUCs was computed with DeLong’s method.

### Identification of substitutes for Stanford11

The list of substitutable genes in Stanford11 was generated based on gene ontology biological processes (GOBPs). We first analyzed the biological functions associated with the Stanford82 genes through functional enrichment analyses of GOBPs using DAVID (Database for Annotation Visualization and Integrated Discovery)^22^. We used GO Direct category which provides GO mappings directly annotated by the source database. The terms with ≥1 genes were selected as Stanford82 associated GOBPs. The GOBPs which included at least one of Stanford11 genes and with at least 2 genes were selected and visualized as a network using Enrichment Map v2.1.0, a Cytoscape plugin^23^. The connected GOBP terms were merged and defined as a single functional category. Genes involved in the merged GOBPs and having the same directional changes were defined as substitutable candidates in the same GOBP.

### Microarray datasets

A total of 12 different microarray datasets were used in two studies by Sweeney *et al*. (Supplementary Table S1)^20^. The first nine datasets are the discovery set that was used to identify Stanford11 and the last three datasets are independent validation sets. The microarray datasets were normalized by the same methods used by Sweeney *et al*. Briefly, Affymetrix datasets were normalized using the Robust Multi-array Average (RMA) or GC-RMA (R package affy)^24^. Agilent and Illumina datasets were background corrected based on normal-exponential convolution model and then between-arrays quantile normalized using R package limma^25^. The mean of multiple probes for common genes was used as the gene expression level after normalization. In the case of GSE74224, there was no probe for KIAA1370 in Stanford11; therefore, only 10 genes were used to compute the performance of Stanford11 for this data.

### Gene substitution and reduction procedure

In order to systematically evaluate the effect of gene substitution and reduction on classification performance, five different procedures were used as follows: (1) substitute one gene at a time; (2) substitute all possible genes; (3) retain one gene for a GOBP where more than two genes are involved; 4) reduce panel by selecting one gene in each GOBP.

### Evaluation of the impact of biological function information

Five different approaches were used to evaluate the impact of biological function based optimization process on classification performance: (1) use the 11 highest correlated genes based on the expression profiles in the Stanford11 panel regardless of their biological functions; (2) use 11 randomly selected genes involved in chemotaxis, adhesion and migration biological function – one of GOBP terms associated with Stanford11; (3) Support Vector Machine with Recursive Feature Elimination (SVM-RFE)^3^; (4) Logistic Regression with Least Absolute Shrinkage and Selection Operator regularization (LR-LASSO)^26^; and (5) *k*-Top Scoring Pairs classifier (*k*TSP) using switchbox R package to identify a small set of paired genes^27^. The AUC of *k*TSP was calculated by defining the number of votes among *k* pairs as a diagnostic score^28^. The 9 microarray data in the discovery set were co-normalized using COmbat CONormalization Using conTrols (COCONUT) R package prior to applying SVM-RFE and LR-LASSO to remove batch effect between different studies^29^. The chemotaxis, adhesion and migration GOBP terms were selected as they contain more than 11 genes that can be fully substituted for Stanford 11 genes. We also tested the classification performance of randomly selected gene sets by generating 100,000 combinations of 11 genes randomly selected from the Stanford82 genes. We analyzed classification performance along with biological processes of the 100,000 gene sets involved in. The top and bottom 250 gene sets in order of performance were selected. The hierarchical clustering was applied to cluster GOBPs based on the number of genes in each GOBP from the top and bottom 250 gene sets.

## Supplementary information

Supplementary information

## Data Availability

The datasets analyzed in this study are available in the Gene Expression Omnibus (GEO) and ArrayExpress repositories. The accession numbers are included in the Supplementary Table S1. The code implementation used for biomarker gene panel optimization was uploaded to a GitHub repository, https://github.com/taekkyun/biomarker-panel-optimization.
