# Supplementary material for: A biological function based biomarker panel optimization process
Source: Sci Rep. 2019 May 14;9:7365. doi: 10.1038/s41598-019-43779-2 (PMC6517383; doi:10.1038/s41598-019-43779-2)
Supplement: Supplementary file 1 — Supplementary information [file 41598_2019_43779_MOESM1_ESM.pdf]

A biological function based biomarker panel optimization process

Min Young Lee, Taek-Kyun Kim, Kathie-Anne Walters, Kai Wang

Supplementary Table S1. The nine discovery and three independent microarray datasets and the performances of Stanford11. Dataset and platform are NCBI GEO accession numbers. AUCs and 95% confidence interval in parentheses were reported by Sweeney et al. CAP is community acquired pneumonia. COPD is chronic obstructive pulmonary disease. ARDS is acute respiratory distress syndrome.

Supplementary Table S2. The list of 82 differentially expressed genes (Stanford82). Summary Effect indicates the direction of fold changes. '1' and '-1' mean up- and downregulation in sepsis compared to SIRS/trauma, respectively.

Supplementary Table S3. The performances of reduced Stanford11 panels by excluding genes not associated to known biological processes or with no substitutable genes. The p-values were calculated by DeLong's test. Yellow in p-value indicates p-value less than 0.05. Orange/green in AUC indicates significantly higher/lower AUC than Stanford11 (P-value ≤ 0.05).

Supplementary Table S4. Alternate optimized six gene panels (n=73). The alternative panels consisted of six genes from the six key biological processes. The 73 panels have higher performance than the lower bound of 95% confidence intervals in all discovery datasets than the original Stanford11. * indicates p-value from DeLong's test in comparison with the Stanford11 less than 0.05.

Supplementary Table S5. The performances of adding RPGRIP1 to the 73 new 6-gene panels. * indicates p-value from DeLong's test in comparison with the new 6 gene panels less than 0.05.

Supplementary Table S1. The nine discovery and three independent microarray datasets and the performances of Stanford11. Dataset and platform are NCBI GEO accession numbers. AUCs and 95% confidence interval in parentheses were reported by Sweeney et al. CAP is community acquired pneumonia. COPD is chronic obstructive pulmonary disease. ARDS is acute respiratory distress syndrome.

	**Dataset**	**Platform**	**Control**	**Case**	**# Control**	**# Case**	**AUC**	**Lower bound of 95% CI**	**Upper bound of 95% CI**
**Discovery set**	GSE28750	GPL570	24 hours post-major surgery	Community-acquired sepsis	11	10	0.96	0.89	1
	GSE32707	GPL10558	Medical ICU ± SIRS, nonseptic	Sepsis, sepsis + ARDS	55	48	0.8	0.71	0.88
	GSE40012	GPL6947	ICU SIRS (66% trauma)	Sepsis from CAP	24	52	0.71	0.59	0.83
	GSE66099	GPL570	Pediatric ICU SIRS	Sepsis and septic shock	30	199	0.79	0.73	0.86
	Glue Grant Buffy coat, day [1–3)	GPL570	Trauma patients without infection	Trauma patients ± 24 hours from diagnosis of infection	65	9	0.91	0.83	1
	Glue Grant Buffy coat, day [3–6)	GPL570	Trauma patients without infection	Trauma patients ± 24 hours from diagnosis of infection	63	17	0.89	0.8	0.98
	Glue Grant Buffy coat, Day [6–10)	GPL570	Trauma patients without infection	Trauma patients ± 24 hours from diagnosis of infection	50	15	0.92	0.84	1
	Glue Grant Buffy coat, day [10–18)	GPL570	Trauma patients without infection	Trauma patients ± 24 hours from diagnosis of infection	22	4	0.85	0.7	1
	Glue Grant Buffy coat, day [18–24)	GPL570	Trauma patients without infection	Trauma patients ± 24 hours from diagnosis of infection	6	4	0.96	0.84	1
**Validation set**	GSE65682	GPL13667	ICU noninfected	CAP	33	101	0.78	0.68	0.87
	GSE74224	GPL5175	Postop	Sepsis	31	74	0.88	0.82	0.95
	E-MEXP-3589	GPL10332	Hospitalized COPD	Infection	14	14	0.74	0.55	0.93

Supplementary Table S2. The list of 82 differentially expressed genes (Stanford82).
Summary Effect indicates the direction of fold changes. '1' and '-1' mean up- and down-regulation in sepsis compared to SIRS/trauma, respectively.

Gene symbol	Summary Effect
ADAMTS3	1
ANKRD22	1
ANXA3	1
AP3B2	1
ARL8A	1
B3GNT8	1
BATF	1
BPI	1
BST1	1
C1orf162	1
C3AR1	1
C9orf103	1
C9orf95	1
CCR1	1
CD177	1
CD63	1
CD82	1
CEACAM1	1
CLEC5A	1
DHRS9	1
EMR1	1
FAM89A	1
FCER1G	1
FCGR1B	1
FES	1
FFAR3	1
FIG4	1
GNA15	1
GPR84	1
HK3	1
HP	1
IL10	1
IL18R1	1
KCNE1	1
LCN2	1
LIN7A	1
OSCAR	1
OSTalpha	1
P2RX1	1
PADI2	1
PECR	1
PLAC8	1
PLB1	1
PNPLA1	1
PPM1M	1
PSTPIP2	1
RETN	1
RGL4	1
S100A12	1
SEPHS2	1
SETD8	1
SGSH	1
SIGLEC9	1
SLC26A8	1
SPPL2A	1
SQRDL	1
TCN1	1
ZDHHC19	1
ZDHHC3	1
ARHGEF18	-1
CACNA2D3	-1
CNNM3	-1
GLO1	-1
GRAMD1C	-1
HACL1	-1
HLA-DPB1	-1
KIAA1370	-1
KLHDC2	-1
METAP1	-1
MRPS35	-1
MTCH1	-1
NOC3L	-1
ODC1	-1
PRKRIR	-1
RPGRIP1	-1
RPUSD4	-1
SETD1B	-1
TBC1D4	-1
TGFBI	-1
TOMM20	-1
UBE2Q2	-1
WDR75	-1

Supplementary Table S3. The performances of reduced Stanford11 panels by excluding genes not associated to known biological processes or with no substitutable genes. The p-values were calculated by DeLong's test. Yellow in p-value indicates p-value less than 0.05. Orange/green in AUC indicates significantly higher/lower AUC than Stanford11 (P-value ≤ 0.05).

		AUC				P-value		
	**Dataset**	Stanford11	Exclude RPGRIP1	Exclude both KIAA1370 and ZDHHC19	Exclude all RPGRIP1, KIAA1370, ZDHHC19	Exclude RPGRIP1	Exclude both KIAA1370 and ZDHHC19	Exclude all RPGRIP1, KIAA1370, ZDHHC19
**Discovery set**	GSE28750	0.9636	0.9636	0.8273	0.7818	1	0.1	0.0672
	GSE32707	0.7962	0.7894	0.7894	0.7678	0.4702	0.5455	0.0715
	GSE40012	0.7091	0.7147	0.7588	0.774	0.7457	0.0174	0.0123
	GSE66099	0.7948	0.8028	0.799	0.8054	0.044	0.7218	0.3916
	Glue Grant Buffy coat, day [1–3)	0.9145	0.9179	0.8632	0.865	0.2997	0.044	0.0434
	Glue Grant Buffy coat, day [3–6)	0.8898	0.8693	0.8898	0.8665	0.1139	1	0.2353
	Glue Grant Buffy coat, Day [6–10)	0.9213	0.9107	0.8947	0.8613	0.502	0.2846	0.1136
	Glue Grant Buffy coat, day [10–18)	0.8523	0.8295	0	0.7159	0.3865	0.8076	0.0784
	Glue Grant Buffy coat, day [18–24)	0.9583	0.875	0.9583	0.875	0.398	1	0.398
**Validation set**	GSE65682	0.7792	0.7843	0.7384	0.7459	0.3798	0.0118	0.398
	GSE74224	0.8814	0.8483	0.8932	0.8544	0.0018	0.0838	0.0186
	E-MEXP-3589	0.7398	0.7449	0.7296	0.7602	0.9025	0.6772	0.6443

Supplementary Table S4. Alternate optimized six gene panels (n=73). The alternative panels consisted of six genes from the six key biological processes. The 73 panels have higher performance than the lower bound of 95% confidence intervals in all discovery datasets than the original Stanford11. * indicates p-value from DeLong's test in comparison with the Stanford11 less than 0.05.

	Chemotaxis, adhesion, migration	Antigen processing, immune response	Transcription by RNA pol II	Platelet activation	Apoptosis	Metabolism	Discovery set									Validation set		
Panel index	Gene1	Gene2	Gene3	Gene4	Gene5	Gene6	GSE28750	GSE32707	GSE40012	GSE66099	Glue Grant Buffy coat, day [1–3)	Glue Grant Buffy coat, day [3–6)	Glue Grant Buffy coat, Day [6–10)	Glue Grant Buffy coat, day [10–18)	Glue Grant Buffy coat, day [18–24)	GSE65682	GSE74224	E-MEXP-3589
1	ADAMTS3	HLA-DPB1	BATF	FCER1G	ARHGEF18	C9orf95	0.8909	0.7648	0.6354	0.7648	0.8359*	0.8711	0.9400	0.7386	0.9583	0.7789	0.7912*	0.5612
2	ADAMTS3	HLA-DPB1	BATF	FCER1G	MTCH1	C9orf95	0.9182	0.7602	0.6587	0.7665	0.8513*	0.8711	0.9360	0.7386	0.9583	0.8119	0.8156*	0.6378
3	CCR1	HLA-DPB1	BATF	GPR84	MTCH1	C9orf103	0.8909	0.7348*	0.6338*	0.7372*	0.8462*	0.8936	0.9600	0.8182	0.9167	0.8218	0.8553	0.6378
4	CCR1	HLA-DPB1	BATF	FCER1G	ARHGEF18	C9orf103	0.9000	0.7117*	0.6042*	0.7504*	0.8735	0.8627	0.9507	0.8750	0.9167	0.7951	0.8535	0.6378
5	CCR1	HLA-DPB1	BATF	FCER1G	ARHGEF18	C9orf95	0.9364	0.7250*	0.6795	0.7786	0.8650*	0.8655	0.9587	0.8295	0.9167	0.7840	0.8435	0.5969
6	CCR1	HLA-DPB1	BATF	FCER1G	MTCH1	C9orf95	0.9455	0.7269*	0.7003	0.7826	0.8667*	0.8861	0.9640	0.8523	0.9167	0.8128	0.8588	0.6684
7	CCR1	HLA-DPB1	BATF	C3AR1	ARHGEF18	C9orf95	0.8909	0.7568	0.6995	0.7896	0.8410*	0.8768	0.9387	0.8750	0.9583	0.7807	0.8854	0.6633
8	CCR1	HLA-DPB1	BATF	C3AR1	MTCH1	C9orf103	0.9000	0.7227*	0.6675	0.7742	0.8821	0.8814	0.9400	0.8750	0.9167	0.8170	0.9058	0.6990
9	CCR1	HLA-DPB1	BATF	C3AR1	MTCH1	C9orf95	0.9091	0.7542	0.7204	0.7940	0.8410*	0.8945	0.9440	0.8409	0.9583	0.8095	0.8967	0.6786
10	CCR1	HLA-DPB1	BATF	GNA15	MTCH1	C9orf95	0.9545	0.7352*	0.7260	0.8062	0.8342*	0.9094	0.9627	0.8636	0.9167	0.8080	0.8893	0.6378
11	CD177	HLA-DPB1	BATF	GPR84	MTCH1	C9orf95	0.8909	0.7470*	0.6274*	0.739*	0.8615	0.8534	0.9373	0.7955	1.0000	0.7933	0.8191*	0.6327
12	CD177	HLA-DPB1	BATF	FCER1G	ARHGEF18	C9orf95	0.9182	0.7159*	0.6050*	0.7653	0.8906	0.8142	0.9293	0.8182	1.0000	0.7762	0.8178*	0.6429
13	CD177	HLA-DPB1	BATF	FCER1G	MTCH1	C9orf95	0.9273	0.7182*	0.6242*	0.7635	0.8974	0.8161	0.9307	0.8182	1.0000	0.8032	0.8304	0.6378
14	CD177	HLA-DPB1	BATF	C3AR1	MTCH1	C9orf95	0.8909	0.7458*	0.6442*	0.7784	0.8838	0.8366	0.9227	0.8295	1.0000	0.8014	0.8827	0.6684
15	CD177	HLA-DPB1	BATF	GNA15	ARHGEF18	C9orf95	0.9273	0.7379*	0.6202*	0.7822	0.8718	0.8403	0.9413	0.8409	1.0000	0.7699	0.8413	0.6429
16	CD177	HLA-DPB1	BATF	GNA15	MTCH1	C9orf95	0.9455	0.7333*	0.6378*	0.7874	0.8752	0.8450	0.9507	0.8295	1.0000	0.7972	0.8570	0.6531
17	CD63	HLA-DPB1	PLAC8	FCER1G	MTCH1	C9orf95	0.9000	0.7231*	0.7244	0.7387*	0.8957	0.8973	0.9067	0.7500	0.9167	0.8158	0.9128	0.6122
18	CD63	HLA-DPB1	PLAC8	GNA15	ARHGEF18	C9orf95	0.9273	0.7163*	0.7372	0.7611	0.8427	0.9328*	0.9307	0.7955	1.0000	0.7852	0.9220	0.5663
19	CD63	HLA-DPB1	PLAC8	GNA15	MTCH1	C9orf95	0.9636	0.7186*	0.7620	0.7670	0.8444	0.9300	0.9427	0.7727	1.0000	0.8107	0.9333*	0.5918
20	CD63	HLA-DPB1	BATF	GPR84	ARHGEF18	C9orf95	0.8909	0.7496*	0.6587	0.7466*	0.8530	0.8758	0.9320	0.7955	1.0000	0.7705	0.8400	0.5969
21	CD63	HLA-DPB1	BATF	GPR84	MTCH1	C9orf103	0.8909	0.7223*	0.6386	0.7328*	0.8769	0.8739	0.9453	0.7841	0.8750	0.8116	0.8505	0.6327
22	CD63	HLA-DPB1	BATF	GPR84	MTCH1	C9orf95	0.8909	0.7481*	0.6803	0.7497*	0.8547	0.8711	0.9427	0.7841	1.0000	0.7993	0.8553	0.6071
23	CD63	HLA-DPB1	BATF	FCER1G	ARHGEF18	C9orf103	0.8909	0.7208*	0.6090*	0.7474*	0.8974	0.8478	0.9387	0.7955	0.8750	0.7942	0.8492	0.6224
24	CD63	HLA-DPB1	BATF	FCER1G	ARHGEF18	C9orf95	0.9000	0.7420	0.6771	0.7784	0.8957	0.8347	0.9440	0.7273	1.0000	0.7780	0.8566	0.6122
25	CD63	HLA-DPB1	BATF	FCER1G	MTCH1	C9orf103	0.9091	0.7231*	0.6346*	0.7422*	0.9060	0.8609	0.9387	0.7614	0.8750	0.8179	0.8636	0.6684
26	CD63	HLA-DPB1	BATF	FCER1G	MTCH1	C9orf95	0.9273	0.7424	0.6979	0.7789	0.9043	0.8478	0.9400	0.7386	0.9583	0.8107	0.8705	0.6276
27	CD63	HLA-DPB1	BATF	C3AR1	MTCH1	C9orf95	0.8909	0.7451	0.7147	0.7953	0.8838	0.8609	0.9227	0.7614	1.0000	0.8080	0.9067	0.6633
28	CD63	HLA-DPB1	BATF	GNA15	ARHGEF18	C9orf95	0.9182	0.7322	0.7188	0.7963	0.8598	0.8702	0.9520	0.7955	0.9583	0.7726	0.8788	0.5969
29	CD63	HLA-DPB1	BATF	GNA15	MTCH1	C9orf95	0.9455	0.7299*	0.7324	0.8005	0.8701	0.8805	0.9547	0.7727	1.0000	0.8029	0.8915	0.6378
30	EMR1	HLA-DPB1	BATF	FCER1G	ARHGEF18	C9orf95	0.9182	0.7216*	0.6506	0.7658	0.8667	0.8581	0.9440	0.8636	1.0000	0.7768	0.8483	0.6071
31	EMR1	HLA-DPB1	BATF	FCER1G	MTCH1	C9orf103	0.8909	0.7117*	0.5962*	0.7353*	0.8632	0.8599	0.9453	0.8636	0.8750	0.8161	0.8496	0.6429
32	EMR1	HLA-DPB1	BATF	FCER1G	MTCH1	C9orf95	0.9182	0.7159*	0.6675	0.7675	0.8701	0.8646	0.9507	0.8636	1.0000	0.8137	0.8596	0.6071
33	EMR1	HLA-DPB1	BATF	C3AR1	ARHGEF18	C9orf95	0.8909	0.7398*	0.6707	0.7791	0.8462	0.8665	0.9227	0.8750	1.0000	0.7795	0.8867	0.6327
34	EMR1	HLA-DPB1	BATF	C3AR1	MTCH1	C9orf95	0.8909	0.7341*	0.6931	0.7851	0.8444	0.8702	0.9333	0.8409	1.0000	0.8149	0.8963	0.6582
35	EMR1	HLA-DPB1	BATF	GNA15	MTCH1	C9orf95	0.9273	0.7159*	0.6915	0.7844	0.8342	0.8833	0.9467	0.8523	1.0000	0.8044	0.8836	0.6071
36	FCER1G	HLA-DPB1	PLAC8	C3AR1	MTCH1	C9orf95	0.9000	0.7273*	0.7356	0.7628	0.8615*	0.8599	0.8747	0.8068	1.0000	0.8080	0.9241	0.6531
37	FCER1G	HLA-DPB1	PLAC8	GNA15	ARHGEF18	C9orf95	0.9182	0.7330	0.7220	0.7688	0.8496	0.9104	0.9173	0.8182	1.0000	0.7861	0.9098	0.5408
38	FCER1G	HLA-DPB1	PLAC8	GNA15	MTCH1	C9orf95	0.9545	0.7322*	0.7460	0.7719	0.8547	0.9188	0.9293	0.7841	1.0000	0.8092	0.9185	0.5816*
39	FCER1G	HLA-DPB1	BATF	GPR84	ARHGEF18	C9orf95	0.8909	0.7508	0.6587	0.7521*	0.8598	0.8609	0.9307	0.8182	1.0000	0.7747	0.8147*	0.5816
40	FCER1G	HLA-DPB1	BATF	GPR84	MTCH1	SEPHS2	0.8909	0.7337*	0.6234*	0.7437*	0.8359*	0.8459	0.9360	0.8068	1.0000	0.8038	0.8095*	0.6122
41	FCER1G	HLA-DPB1	BATF	GPR84	MTCH1	C9orf95	0.8909	0.7424*	0.6755	0.7514*	0.8615	0.8665	0.9387	0.7727	0.9583	0.8041	0.833*	0.6071
42	FCER1G	HLA-DPB1	BATF	C3AR1	MTCH1	SEPHS2	0.9000	0.7273*	0.6619	0.7995	0.8667	0.8030*	0.9187	0.8409	1.0000	0.8086	0.8867	0.6582
43	FCER1G	HLA-DPB1	BATF	C3AR1	MTCH1	C9orf95	0.9000	0.7413	0.7083	0.7980	0.8889	0.8413	0.9227	0.8068	1.0000	0.8071	0.8963	0.6480
44	FCER1G	HLA-DPB1	BATF	GNA15	ARHGEF18	C9orf95	0.9455	0.7413	0.6947	0.8027	0.8735	0.8553	0.9520	0.7841	0.9583	0.7699	0.8640	0.5867
45	FCER1G	HLA-DPB1	BATF	GNA15	MTCH1	SEPHS2	0.9727	0.7242*	0.6747	0.7982	0.8359*	0.8329	0.9480	0.8409	1.0000	0.8077	0.8614	0.6224
46	FCER1G	HLA-DPB1	BATF	GNA15	MTCH1	C9orf95	0.9818	0.7352	0.7123	0.8040	0.8872	0.8599	0.9547	0.7841	0.9583	0.8026	0.8749	0.6122
47	OSTalpha	HLA-DPB1	BATF	GNA15	ARHGEF18	C9orf95	0.8909	0.7701	0.6338*	0.7496*	0.8940	0.8852	0.9280	0.8409	0.9167	0.7690	0.8147*	0.5918
48	OSTalpha	HLA-DPB1	BATF	GNA15	MTCH1	SEPHS2	0.8909	0.7295*	0.6242*	0.7367*	0.8855	0.8693	0.9320	0.8523	0.8750	0.8065	0.7960*	0.6276
49	OSTalpha	HLA-DPB1	BATF	GNA15	MTCH1	C9orf95	0.9091	0.7640	0.6474	0.7487*	0.8991	0.8805	0.9307	0.8295	0.8750	0.8035	0.8326	0.6224
50	SIGLEC9	HLA-DPB1	BATF	GNA15	MTCH1	C9orf95	0.9455	0.7799	0.6907	0.7506*	0.8906	0.8123*	0.9320	0.8182	1.0000	0.8125	0.8745	0.6582
51	ANXA3	HLA-DPB1	BATF	GNA15	MTCH1	C9orf95	0.9091	0.7152*	0.6530	0.7930	0.8581	0.8702	0.9373	0.8182	1.0000	0.7993	0.8383	0.6531
52	FES	HLA-DPB1	PLAC8	GPR84	MTCH1	C9orf95	0.8909	0.7583	0.7027	0.7320*	0.8427	0.8553	0.9160	0.7841	1.0000	0.7939	0.8758	0.6020
53	FES	HLA-DPB1	PLAC8	FCER1G	MTCH1	C9orf95	0.9091	0.7318*	0.7204	0.7521	0.8906	0.8385	0.8920	0.7841	1.0000	0.8089	0.8945	0.6173
54	FES	HLA-DPB1	PLAC8	GNA15	ARHGEF18	C9orf103	0.8909	0.7292*	0.6643	0.7441*	0.8701	0.8749	0.9187	0.8409	0.8750	0.7870	0.9098	0.6122
55	FES	HLA-DPB1	PLAC8	GNA15	ARHGEF18	C9orf95	0.9000	0.7470	0.7364	0.7735	0.8427	0.8693	0.9147	0.8182	0.9583	0.7768	0.9102	0.5867
56	FES	HLA-DPB1	PLAC8	GNA15	MTCH1	C9orf95	0.9545	0.7428	0.7500	0.7760	0.8513	0.8655	0.9120	0.8068	0.9583	0.8005	0.9150	0.6071
57	FES	HLA-DPB1	BATF	GPR84	MTCH1	C9orf95	0.8909	0.7765	0.6851	0.7553*	0.8479	0.8487*	0.9227	0.7955	1.0000	0.7999	0.8309*	0.6327
58	FES	HLA-DPB1	BATF	FCER1G	ARHGEF18	C9orf103	0.8909	0.7348*	0.6050*	0.7513	0.8855	0.8114*	0.9347	0.7841	0.8750	0.7897	0.8069*	0.6633
59	FES	HLA-DPB1	BATF	FCER1G	MTCH1	C9orf103	0.9000	0.7341*	0.6258*	0.7497	0.8889	0.8226*	0.9333	0.8068	0.8750	0.8158	0.8291	0.6633
60	FES	HLA-DPB1	BATF	C3AR1	MTCH1	C9orf95	0.9000	0.7799	0.7115	0.7970	0.8667	0.8161*	0.9160	0.7841	1.0000	0.8026	0.8806	0.6786
61	FES	HLA-DPB1	BATF	GNA15	ARHGEF18	C9orf95	0.9273	0.7742	0.7011	0.7968	0.8564	0.8226	0.9320	0.7955	0.9583	0.7672	0.8470	0.6173
62	FES	HLA-DPB1	BATF	GNA15	MTCH1	C9orf95	0.9455	0.7670	0.7260	0.8022	0.8598	0.8245	0.9427	0.7841	0.9583	0.7990	0.8627	0.6480
63	S100A12	HLA-DPB1	PLAC8	FCER1G	MTCH1	C9orf95	0.9000	0.7174*	0.6907	0.7367*	0.8855	0.8926	0.9053	0.7955	0.9583	0.8155	0.8718	0.6122
64	S100A12	HLA-DPB1	BATF	GPR84	ARHGEF18	C9orf95	0.8909	0.7367*	0.6482	0.7476*	0.8479*	0.8768	0.9280	0.7955	1.0000	0.7717	0.7951*	0.6122
65	S100A12	HLA-DPB1	BATF	GPR84	MTCH1	C9orf95	0.8909	0.7307*	0.6603	0.7504*	0.8513	0.8758	0.9320	0.7841	1.0000	0.8002	0.8112*	0.6122
66	S100A12	HLA-DPB1	BATF	FCER1G	ARHGEF18	C9orf95	0.9000	0.7280*	0.6482*	0.7782	0.8940	0.8599	0.9507	0.7614	1.0000	0.7786	0.7942*	0.6020
67	S100A12	HLA-DPB1	BATF	FCER1G	MTCH1	C9orf95	0.9182	0.7242*	0.6611	0.7839	0.8957	0.8693	0.9493	0.7500	1.0000	0.8086	0.8134*	0.6378
68	S100A12	HLA-DPB1	BATF	C3AR1	MTCH1	C9orf95	0.9000	0.7311*	0.6939	0.7998	0.8752	0.8730	0.9093	0.7955	1.0000	0.8074	0.8670	0.6531
69	S100A12	HLA-DPB1	BATF	GNA15	ARHGEF18	C9orf95	0.9182	0.7197*	0.6747	0.7963	0.8564	0.8945	0.9453	0.7841	1.0000	0.7726	0.8352	0.6071
70	S100A12	HLA-DPB1	BATF	GNA15	MTCH1	C9orf103	0.9000	0.7106*	0.6202*	0.7693	0.8855	0.9010	0.9547	0.8523	0.8750	0.8128	0.8405	0.6327
71	S100A12	HLA-DPB1	BATF	GNA15	MTCH1	C9orf95	0.9455	0.7201*	0.6947	0.8044	0.8684	0.8973	0.9560	0.8068	1.0000	0.8035	0.8461	0.6276
72	C3AR1	HLA-DPB1	BATF	GNA15	ARHGEF18	C9orf95	0.9182	0.7583	0.7091	0.8092	0.8513	0.8693	0.9187	0.8182	1.0000	0.7678	0.9050	0.6276
73	C3AR1	HLA-DPB1	BATF	GNA15	MTCH1	C9orf95	0.9273	0.7542	0.7228	0.8136	0.8479	0.8711	0.9267	0.8182	1.0000	0.7999	0.9133	0.6480

Supplementary Table S5. The performances of adding RPGRIP1 to the 73 new 6-gene panels. * indicates p-value from DeLong's test in comparison with the new 6 gene panels less than 0.05.

	Chemotaxis, adhesion, migration	Antigen processing, immune response	Transcription by RNA pol II	Platelet activation	Apoptosis	Metabolism		Discovery set									Validation set		
**Panel index**	**Gene1**	**Gene2**	**Gene3**	**Gene4**	**Gene5**	**Gene6**	**Gene7**	**GSE28750**	**GSE32707**	**GSE40012**	**GSE66099**	**Glue Grant Buffy coat, day [1–3)**	**Glue Grant Buffy coat, day [3–6)**	**Glue Grant Buffy coat, Day [6–10)**	**Glue Grant Buffy coat, day [10–18)**	**Glue Grant Buffy coat, day [18–24)**	**GSE65682**	**GSE74224**	**E-MEXP-3589**
1	ADAMTS3	HLA-DPB1	BATF	FCER1G	ARHGEF18	C9orf95	RPGRIP1	0.9182	0.7932	0.6530	0.7554	0.8410	0.8992	0.9440	0.8182	1.0000	0.7525	0.8714*	0.6327
2	ADAMTS3	HLA-DPB1	BATF	FCER1G	MTCH1	C9orf95	RPGRIP1	0.9273	0.7886	0.6659	0.7526	0.8462	0.9085	0.9480	0.8295	1.0000	0.7534	0.8945*	0.6582
3	CCR1	HLA-DPB1	BATF	GPR84	MTCH1	C9orf103	RPGRIP1	0.9182	0.7496	0.6458	0.7302	0.8444	0.9169	0.9507	0.8636	1.0000	0.7594	0.8836*	0.6531
4	CCR1	HLA-DPB1	BATF	FCER1G	ARHGEF18	C9orf103	RPGRIP1	0.9455	0.7261	0.6338	0.7471	0.8735	0.9085*	0.9507	0.8864	0.9583	0.7678	0.8980*	0.6531
5	CCR1	HLA-DPB1	BATF	FCER1G	ARHGEF18	C9orf95	RPGRIP1	0.9364	0.7568	0.7019	0.7744	0.8615	0.8926	0.9627	0.8864	1.0000	0.7432	0.8840*	0.6378
6	CCR1	HLA-DPB1	BATF	FCER1G	MTCH1	C9orf95	RPGRIP1	0.9545	0.7534	0.7179	0.7764	0.8632	0.9029	0.9680	0.8864	1.0000	0.7390	0.8993*	0.6429
7	CCR1	HLA-DPB1	BATF	C3AR1	ARHGEF18	C9orf95	RPGRIP1	0.9091	0.7758	0.7171	0.7814	0.8376	0.9001	0.9453	0.8864	1.0000	0.7405	0.9189*	0.6735
8	CCR1	HLA-DPB1	BATF	C3AR1	MTCH1	C9orf103	RPGRIP1	0.9273	0.7508	0.6819	0.7640	0.8667	0.9094	0.9413	0.8864	1.0000	0.7627	0.9381*	0.6735
9	CCR1	HLA-DPB1	BATF	C3AR1	MTCH1	C9orf95	RPGRIP1	0.9273	0.7742	0.7308	0.7843	0.8376	0.9085	0.9480	0.8864	1.0000	0.7300	0.9329*	0.6888
10	CCR1	HLA-DPB1	BATF	GNA15	MTCH1	C9orf95	RPGRIP1	0.9455	0.7652	0.7340	0.7941	0.8308	0.9244	0.9667	0.9091	1.0000	0.7348	0.9289*	0.6071
11	CD177	HLA-DPB1	BATF	GPR84	MTCH1	C9orf95	RPGRIP1	0.9000	0.7470	0.6546	0.7310	0.8684	0.8786	0.9400	0.8295	1.0000	0.7378	0.8540*	0.6378
12	CD177	HLA-DPB1	BATF	FCER1G	ARHGEF18	C9orf95	RPGRIP1	0.9182	0.7424	0.6474	0.7605	0.8957	0.8403	0.9280	0.8182	1.0000	0.7474	0.8614*	0.6786
13	CD177	HLA-DPB1	BATF	FCER1G	MTCH1	C9orf95	RPGRIP1	0.9455	0.7436	0.6563	0.7576	0.9009	0.8459*	0.9360	0.8182	1.0000	0.7489	0.8727*	0.6684
14	CD177	HLA-DPB1	BATF	C3AR1	MTCH1	C9orf95	RPGRIP1	0.9091	0.7625	0.6675	0.7709	0.8923	0.8590	0.9293	0.8295	1.0000	0.7444	0.9176*	0.6786
15	CD177	HLA-DPB1	BATF	GNA15	ARHGEF18	C9orf95	RPGRIP1	0.9455	0.7564	0.6563	0.7787	0.8752	0.8646	0.9600	0.8409	1.0000	0.7387	0.8862*	0.6684
16	CD177	HLA-DPB1	BATF	GNA15	MTCH1	C9orf95	RPGRIP1	0.9727	0.7538	0.6707	0.7784	0.8821	0.8702	0.9640	0.8409	1.0000	0.7399	0.8997*	0.6735
17	CD63	HLA-DPB1	PLAC8	FCER1G	MTCH1	C9orf95	RPGRIP1	0.9182	0.7364	0.7220	0.7266	0.8923	0.9197	0.9200	0.8409	1.0000	0.7579	0.9394*	0.6173
18	CD63	HLA-DPB1	PLAC8	GNA15	ARHGEF18	C9orf95	RPGRIP1	0.9545	0.7201	0.7356	0.7596	0.8427	0.9458	0.9387	0.8523	1.0000	0.7477	0.9507*	0.6173
19	CD63	HLA-DPB1	PLAC8	GNA15	MTCH1	C9orf95	RPGRIP1	0.9636	0.7277	0.7388	0.7611	0.8496	0.9524	0.9440	0.8295	1.0000	0.7477	0.9568*	0.6173
20	CD63	HLA-DPB1	BATF	GPR84	ARHGEF18	C9orf95	RPGRIP1	0.8909	0.7595	0.6803	0.7427	0.8547	0.8982	0.9400	0.8068	1.0000	0.7270	0.8836*	0.6531
21	CD63	HLA-DPB1	BATF	GPR84	MTCH1	C9orf103	RPGRIP1	0.9000	0.7242	0.6426	0.7233	0.8735	0.9020	0.9467	0.8068	0.9583	0.7489	0.8849*	0.6224
22	CD63	HLA-DPB1	BATF	GPR84	MTCH1	C9orf95	RPGRIP1	0.8909	0.7617	0.6907	0.7430	0.8564	0.8982	0.9440	0.7955	1.0000	0.7279	0.8958*	0.6378
23	CD63	HLA-DPB1	BATF	FCER1G	ARHGEF18	C9orf103	RPGRIP1	0.9091	0.7258	0.6378	0.7395	0.8957	0.8898	0.9413	0.8182	0.9583	0.7621	0.9037*	0.6378
24	CD63	HLA-DPB1	BATF	FCER1G	ARHGEF18	C9orf95	RPGRIP1	0.9273	0.7545	0.6907	0.7704	0.8838	0.8525	0.9493	0.8182*	1.0000	0.7405	0.9067*	0.6786
25	CD63	HLA-DPB1	BATF	FCER1G	MTCH1	C9orf103	RPGRIP1	0.9364	0.7250	0.6482	0.7327	0.8991	0.8908	0.9453	0.8295	0.9583	0.7609	0.9102*	0.6480
26	CD63	HLA-DPB1	BATF	FCER1G	MTCH1	C9orf95	RPGRIP1	0.9364	0.7542	0.6995	0.7717	0.8923	0.8665	0.9560	0.8068*	1.0000	0.7372	0.9167*	0.6633
27	CD63	HLA-DPB1	BATF	C3AR1	MTCH1	C9orf95	RPGRIP1	0.9091	0.7678	0.7204	0.7841	0.8718	0.8730	0.9413	0.8295	1.0000	0.7285	0.9446*	0.6990
28	CD63	HLA-DPB1	BATF	GNA15	ARHGEF18	C9orf95	RPGRIP1	0.9273	0.7511	0.7083	0.7901	0.8632	0.8758	0.9613	0.8295	1.0000	0.7303	0.9285*	0.6633
29	CD63	HLA-DPB1	BATF	GNA15	MTCH1	C9orf95	RPGRIP1	0.9364	0.7519	0.7196	0.7871	0.8650	0.8861	0.9653	0.8182	1.0000	0.7300	0.9416*	0.6429
30	EMR1	HLA-DPB1	BATF	FCER1G	ARHGEF18	C9orf95	RPGRIP1	0.9182	0.7470	0.6691	0.7580	0.8650	0.8908	0.9507	0.8864	1.0000	0.7378	0.8806*	0.6276
31	EMR1	HLA-DPB1	BATF	FCER1G	MTCH1	C9orf103	RPGRIP1	0.9545	0.7201	0.3814	0.7256	0.8615	0.8898	0.9467	0.8977	0.9583	0.7642	0.8840*	0.6531
32	EMR1	HLA-DPB1	BATF	FCER1G	MTCH1	C9orf95	RPGRIP1	0.9455	0.7420	0.6779	0.7601	0.8667	0.8954	0.9547	0.8977	1.0000	0.7366	0.8923*	0.6378
33	EMR1	HLA-DPB1	BATF	C3AR1	ARHGEF18	C9orf95	RPGRIP1	0.9091	0.7648	0.6803	0.7735	0.8530	0.8908	0.9307	0.9091	1.0000	0.7363	0.9185*	0.6837
34	EMR1	HLA-DPB1	BATF	C3AR1	MTCH1	C9orf95	RPGRIP1	0.9182	0.7663	0.6979	0.7760	0.8530	0.8992	0.9320	0.8977	1.0000	0.7330	0.9289*	0.6837
35	EMR1	HLA-DPB1	BATF	GNA15	MTCH1	C9orf95	RPGRIP1	0.9273	0.7489	0.6875	0.7765	0.8393	0.9225	0.9520	0.9318	1.0000	0.7267	0.9180*	0.6071
36	FCER1G	HLA-DPB1	PLAC8	C3AR1	MTCH1	C9orf95	RPGRIP1	0.9182	0.7462	0.7412	0.7571	0.8581	0.8936*	0.9013	0.8295	1.0000	0.7465	0.9429*	0.6888
37	FCER1G	HLA-DPB1	PLAC8	GNA15	ARHGEF18	C9orf95	RPGRIP1	0.9455	0.7500	0.7300	0.7675	0.8530	0.9328	0.9293	0.8636	1.0000	0.7495	0.9307*	0.5969
38	FCER1G	HLA-DPB1	PLAC8	GNA15	MTCH1	C9orf95	RPGRIP1	0.9636	0.7481	0.7396	0.7685	0.8462	0.9430	0.9373	0.8523	1.0000	0.7486	0.9425*	0.6122
39	FCER1G	HLA-DPB1	BATF	GPR84	ARHGEF18	C9orf95	RPGRIP1	0.8909	0.7708	0.6731	0.7462	0.8547	0.8852	0.9347	0.8068	1.0000	0.7270	0.8605*	0.6224
40	FCER1G	HLA-DPB1	BATF	GPR84	MTCH1	SEPHS2	RPGRIP1	0.8909	0.7462	0.6410	0.7410	0.8427	0.8618	0.9400	0.8409	1.0000	0.7249	0.8492*	0.6378
41	FCER1G	HLA-DPB1	BATF	GPR84	MTCH1	C9orf95	RPGRIP1	0.9091	0.7693	0.6811	0.7467	0.8598	0.8936	0.9360	0.7841	1.0000	0.7246	0.8701*	0.6378
42	FCER1G	HLA-DPB1	BATF	C3AR1	MTCH1	SEPHS2	RPGRIP1	0.9182	0.7428	0.6827	0.7906	0.8615	0.8301	0.9307	0.8750	1.0000	0.7291	0.9355*	0.6990
43	FCER1G	HLA-DPB1	BATF	C3AR1	MTCH1	C9orf95	RPGRIP1	0.9000	0.7674	0.7196	0.7898	0.8752	0.8721	0.9293	0.8523	1.0000	0.7276	0.9307*	0.6990
44	FCER1G	HLA-DPB1	BATF	GNA15	ARHGEF18	C9orf95	RPGRIP1	0.9455	0.7659	0.7035	0.7960	0.8718	0.8665	0.9547	0.8523	1.0000	0.7336	0.9106*	0.6633
45	FCER1G	HLA-DPB1	BATF	GNA15	MTCH1	SEPHS2	RPGRIP1	0.9727	0.7383	0.6659	0.7879	0.8427	0.8497	0.9613	0.8750	1.0000	0.7267	0.9124*	0.6429
46	FCER1G	HLA-DPB1	BATF	GNA15	MTCH1	C9orf95	RPGRIP1	0.9636	0.7598	0.7155	0.7925	0.8803	0.8814	0.9627	0.8409	1.0000	0.7324	0.9220*	0.6378
47	OSTalpha	HLA-DPB1	BATF	GNA15	ARHGEF18	C9orf95	RPGRIP1	0.9273	0.7780	0.6522	0.7374	0.8991	0.9076	0.9400	0.8636	0.9583	0.7459	0.8779*	0.6429
48	OSTalpha	HLA-DPB1	BATF	GNA15	MTCH1	SEPHS2	RPGRIP1	0.9364	0.7538	0.6314	0.7231	0.8906	0.9010*	0.9400	0.9091	0.9583	0.7459	0.8710*	0.6429
49	OSTalpha	HLA-DPB1	BATF	GNA15	MTCH1	C9orf95	RPGRIP1	0.9364	0.7788	0.6595	0.7355	0.9026	0.9020	0.9387	0.8750	0.9583	0.7462	0.8897*	0.6582
50	SIGLEC9	HLA-DPB1	BATF	GNA15	MTCH1	C9orf95	RPGRIP1	0.9455	0.8000	0.6931	0.7352	0.8821	0.8263	0.9373	0.8636	1.0000	0.7537	0.9259*	0.6837
51	ANXA3	HLA-DPB1	BATF	GNA15	MTCH1	C9orf95	RPGRIP1	0.9182	0.7470	0.6859	0.7817	0.8564	0.8964	0.9467	0.8750	1.0000	0.7375	0.8823*	0.6786
52	FES	HLA-DPB1	PLAC8	GPR84	MTCH1	C9orf95	RPGRIP1	0.9000	0.7803	0.7003	0.7286	0.8564	0.8786	0.9107	0.8182	1.0000	0.7510	0.9032*	0.6480
53	FES	HLA-DPB1	PLAC8	FCER1G	MTCH1	C9orf95	RPGRIP1	0.9273	0.7519	0.7091	0.7466	0.8838	0.8870	0.9173	0.8295	1.0000	0.7615	0.9316*	0.6582
54	FES	HLA-DPB1	PLAC8	GNA15	ARHGEF18	C9orf103	RPGRIP1	0.9273	0.7394	0.6635	0.7332	0.8667	0.9160	0.9387	0.8750	0.9583	0.7627	0.9394*	0.6020
55	FES	HLA-DPB1	PLAC8	GNA15	ARHGEF18	C9orf95	RPGRIP1	0.9364	0.7727	0.7212	0.7643	0.8530	0.9029*	0.9293	0.8636	1.0000	0.7510	0.9407*	0.6378
56	FES	HLA-DPB1	PLAC8	GNA15	MTCH1	C9orf95	RPGRIP1	0.9455	0.7686	0.7356	0.7662	0.8530	0.9076*	0.9400	0.8750	1.0000	0.7492	0.9494*	0.6429
57	FES	HLA-DPB1	BATF	GPR84	MTCH1	C9orf95	RPGRIP1	0.8909	0.7917	0.6819	0.7484	0.8462	0.8777	0.9387	0.8295	1.0000	0.7300	0.8710*	0.6786
58	FES	HLA-DPB1	BATF	FCER1G	ARHGEF18	C9orf103	RPGRIP1	0.9000	0.7500	0.6298	0.7462	0.8821	0.8543	0.9413	0.8409	0.9583	0.7606	0.8806*	0.6837
59	FES	HLA-DPB1	BATF	FCER1G	MTCH1	C9orf103	RPGRIP1	0.9364	0.7436	0.6394	0.7430	0.8889	0.8553	0.9453	0.8523	0.9583	0.7615	0.8945*	0.6837
60	FES	HLA-DPB1	BATF	C3AR1	MTCH1	C9orf95	RPGRIP1	0.9091	0.7989	0.7188	0.7824	0.8701	0.8385	0.9373	0.8523	1.0000	0.7249	0.9303*	0.7449
61	FES	HLA-DPB1	BATF	GNA15	ARHGEF18	C9orf95	RPGRIP1	0.9273	0.7936	0.7035	0.7901	0.8650	0.8497*	0.9480	0.8409	1.0000	0.7342	0.9089*	0.6939
62	FES	HLA-DPB1	BATF	GNA15	MTCH1	C9orf95	RPGRIP1	0.9455	0.7924	0.7139	0.7899	0.8718	0.8525*	0.9573	0.8409	1.0000	0.7333	0.9250*	0.6837
63	S100A12	HLA-DPB1	PLAC8	FCER1G	MTCH1	C9orf95	RPGRIP1	0.9000	0.7299	0.7011	0.7320	0.8752	0.9346*	0.9147	0.8182	1.0000	0.7600	0.8980*	0.6122
64	S100A12	HLA-DPB1	BATF	GPR84	ARHGEF18	C9orf95	RPGRIP1	0.8909	0.7621	0.6659	0.7420	0.8479	0.9001	0.9293	0.7955	1.0000	0.7261	0.8435*	0.6327
65	S100A12	HLA-DPB1	BATF	GPR84	MTCH1	C9orf95	RPGRIP1	0.8909	0.7583	0.6731	0.7402	0.8513	0.8982	0.9293	0.7841	1.0000	0.7225	0.8548*	0.6378
66	S100A12	HLA-DPB1	BATF	FCER1G	ARHGEF18	C9orf95	RPGRIP1	0.9091	0.7451	0.6787	0.7737	0.8872	0.8852	0.9520	0.8295	1.0000	0.7360	0.8514*	0.6378
67	S100A12	HLA-DPB1	BATF	FCER1G	MTCH1	C9orf95	RPGRIP1	0.9182	0.7390	0.6899	0.7735	0.8957	0.8964	0.9587	0.8068	1.0000	0.7360	0.8710*	0.6327
68	S100A12	HLA-DPB1	BATF	C3AR1	MTCH1	C9orf95	RPGRIP1	0.9000	0.7602	0.6987	0.7881	0.8667	0.8898	0.9307	0.8636*	1.0000	0.7240	0.9106*	0.6888
69	S100A12	HLA-DPB1	BATF	GNA15	ARHGEF18	C9orf95	RPGRIP1	0.9273	0.7527	0.6915	0.7950	0.8581	0.9094	0.9520	0.8523	1.0000	0.7276	0.8888*	0.6327
70	S100A12	HLA-DPB1	BATF	GNA15	MTCH1	C9orf103	RPGRIP1	0.9545	0.7254	0.6514	0.7625	0.8872	0.9374	0.9547	0.8636	0.9583	0.7513	0.8976*	0.6224
71	S100A12	HLA-DPB1	BATF	GNA15	MTCH1	C9orf95	RPGRIP1	0.9364	0.7481	0.7043	0.7953	0.8718	0.9150	0.9560	0.8409	1.0000	0.7243	0.9010*	0.6327
72	C3AR1	HLA-DPB1	BATF	GNA15	ARHGEF18	C9orf95	RPGRIP1	0.9273	0.7856	0.7220	0.8017	0.8513	0.8749	0.9360	0.8864	1.0000	0.7195	0.9381*	0.7143
73	C3AR1	HLA-DPB1	BATF	GNA15	MTCH1	C9orf95	RPGRIP1	0.9455	0.7830	0.7340	0.8030	0.8427	0.8898	0.9373	0.8750	1.0000	0.7177	0.9459*	0.7041
